# Supplementary material for: Lateral pressure equalisation as a principle for designing support surfaces to prevent deep tissue pressure ulcers
Source: PLoS One. 2020 Jan 3;15(1):e0227064. doi: 10.1371/journal.pone.0227064 (PMC6941906; doi:10.1371/journal.pone.0227064)
Supplement: S1 Appendix — (PDF) [file pone.0227064.s001.pdf]

S1 Analysis of pressure distributions

The surface pressure induced when seated on a cushion follows a characteristic shape (Fig 1a): there is a narrow peak directly beneath the ischial tuberosity, occupying only a small region of the contact area. Pressure as a function of angle from the z-axis (Fig 1 b) is adequately described using a gaussian function:

$$P(\theta) = P_V e^{-\frac{(\theta)^2}{2\alpha^2}},\ \theta < \frac{\pi}{2}$$

where θ is the swept angle between the vertical and the surface position, α controls the spread of the curve and P_V is the peak pressure magnitude. Non-linear least squares fitting (using the curve_fit function of the scipy module) was used to fit the parameters for each of the three cushion types (Table 1).

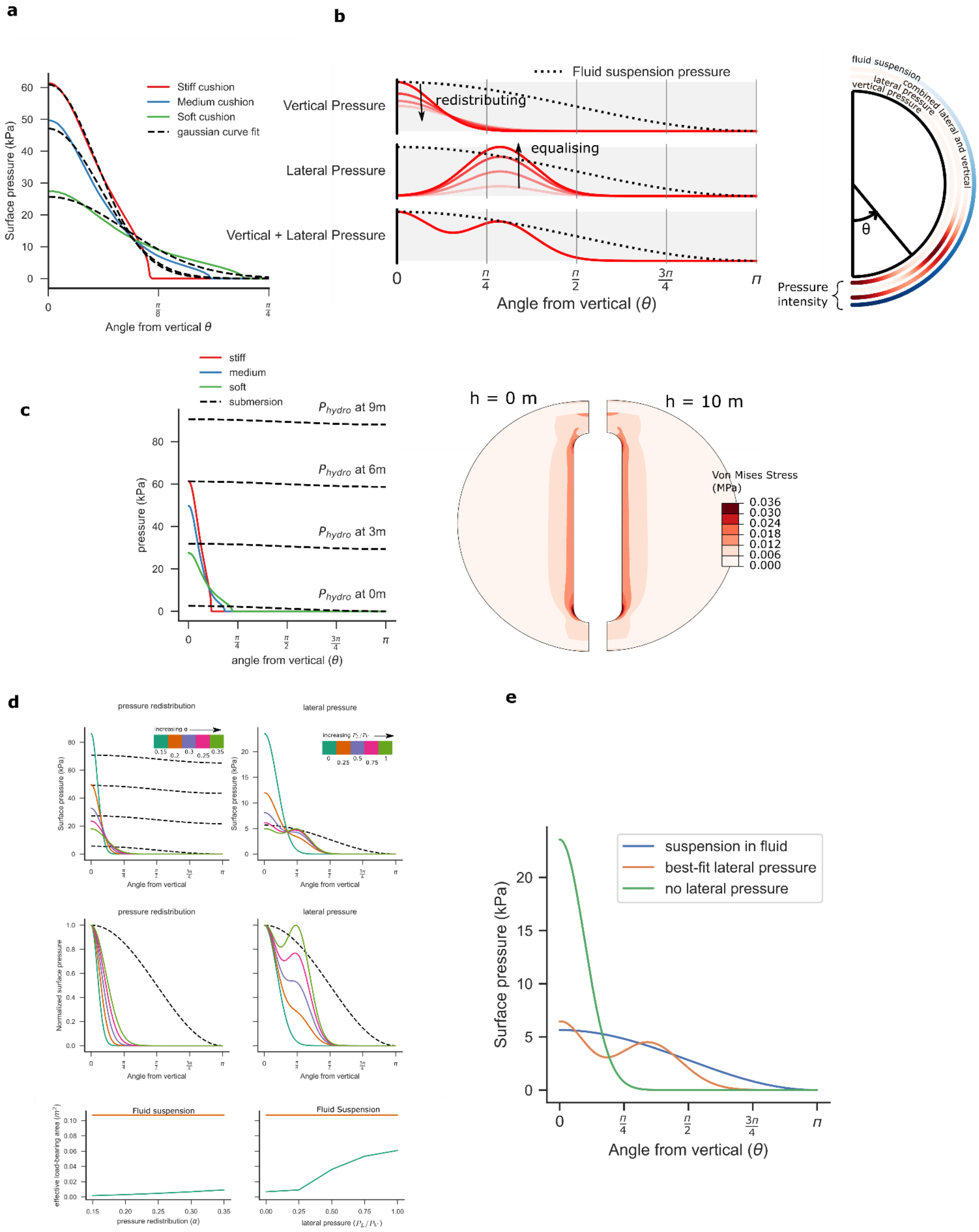
a
Surface pressure (kPa)
Stiff cushion
Medium cushion
Soft cushion
gaussian curve fit
Angle from vertical θ
b
Fluid suspension pressure
Vertical Pressure
redistributing
Lateral Pressure
equalising
Vertical + Lateral Pressure
Angle from vertical (θ)
fluid suspension
combined lateral and vertical
lateral pressure
vertical pressure
θ
Pressure intensity
c
stiff
medium
soft
submersion
P_{hydro} at 9m
P_{hydro} at 6m
P_{hydro} at 3m
P_{hydro} at 0m
pressure (kPa)
angle from vertical (θ)
h = 0 m
h = 10 m
Von Mises Stress (MPa)
0.036
0.030
0.024
0.018
0.012
0.006
0.000
d
pressure redistribution
lateral pressure
increasing α
Surface pressure (kPa)
Angle from vertical
Normalized surface pressure
Fluid suspension
Fluid Suspension
effective load-bearing area (m^2)
pressure redistribution (α)
lateral pressure (P_L/P_V)
e
suspension in fluid
best-fit lateral pressure
no lateral pressure
Surface pressure (kPa)
Angle from vertical (θ)

Fig 1. a Each of the cushions modelled in section 2.2 produces a similar pressure distribution at the skin surface: There is a peak directly beneath the ischial tuberosity ($\theta = 0$), which drops to zero at the periphery of contact. Here, pressure is plotted as a function of the swept angle between the vertical and the position. A gaussian function provides a good fit for this distribution. **b** A schematic showing the lateral and under-body surface pressures. **c** The surface pressure induced when suspended in a fluid (dotted line) is relatively constant from each direction compared to the pressure profile when seated on a cushion (coloured lines). The magnitude of pressure varies substantially with depth, and even at 6 m below the surface, all surfaces are exposed to a pressure greater than that induced by a stiff cushion. When a pressure field representing 10 m submersion is compared to one representing pressure at the surface, the dilatational stress in the tissue increases (from 17kPa at 0m to 243kPa at 10m), but the deviatoric stresses are unchanged. **d** Both pressure re-distribution (increasing α) and applying lateral pressure (increasing P_L/P_V) reduces the peak pressure (top). Applying lateral pressure mimics the hydrostatic loading distribution more effectively than redistributing pressure (middle). The effective load-bearing area (area experiencing more than 50% of the peak pressure value) is increased more with lateral pressure than with re-distribution (bottom). **e** By optimising the magnitude and location of lateral pressure, the pressure profile around the soft tissue can accurately mimic that of suspension in a fluid (hydrostatic pressure).

Table 1. Parameters found through non-linear least square fitting of a gaussian distribution to the surface pressure.

Cushion	$P_V(kPa)$	α (-)
Stiff	60.9	0.170
Medium	47.1	0.184
Soft	25.7	0.272

Pressure distribution while suspended in a fluid

Suspension in a fluid can be regarded as a best-case scenario for load-bearing [20]. In this case, the pressure distribution is given as:

$$P_{Hydro}(\theta) = \rho g(h_0 + r\cos\theta)$$

where ρ is density (997 for water), g = 9.81 , h_0 is the depth of submersion from the fluid surface to the model centre and r is the outer surface radius (130). The shape of the distribution remains constant as depth is increased, while the magnitudes of the pressures increase (Fig 1c). The dilatational stress within the soft tissue increases (from 17kPa when h0=130mm to 243kPa when h0=10130mm). In contrast, deviatoric stresses (von Mises) do not change at all as depth is increased.

Pressure re-distribution vs pressure equalisation

We modelled the introduction of a lateral pressure equalisation device by applying a new gaussian term, offset from vertical by angle θ_0.

$$
\begin{aligned}
P(\theta) &= P_V f(\theta) + P_L g(\theta), \\
f(\theta) &= e^{-\frac{(\theta)^2}{2\alpha^2}},\ \theta < \frac{\pi}{2} \\
g(\theta) &= e^{-\frac{(\theta-\theta_0)^2}{2\beta^2}}
\end{aligned}
$$

In these equations, P_V and P_L are the vertical and lateral pressure peak magnitudes, α and β control their spread, and θ_0 controls the lateral peak location (Fig 1b). Supplementary figure 1d shows that the ideal pressure profile (that of suspension in a fluid) is best achieved by applying lateral pressure. Taken from a different perspective, an objective of support surface design can be seen as trying to maximise the area of contact between the soft tissue and the cushion. Fig 1b shows that applying a small amount of lateral pressure increases the effective load bearing area[1] far more than redistributing under-body pressure.

Scaling pressure distributions

Once the shape of $P(\theta)$ was described from any of the sections above, it was scaled such that it exerted a net force in the y-direction of 200 N, i.e. to support body force W. The force in the z direction acting on an infinitesimal area dS of a sphere of radius r is $dF_z = P(\theta) \cdot \cos(\theta) dS$,

dS can be parameterised as $dS = r^2 \sin(\theta) d\theta d\phi$, where θ is the polar angle and ϕ the azimuthal angle. The total force in the z direction is then:

$$F_z = \int_0^{2\pi} \int_0^{\pi} r^2\, P(\theta) \cdot \cos\theta \sin\theta d\theta d\phi = \pi r^2 \int_0^{\pi} P\,(\theta) \sin(2\theta) d\theta.$$

[1] This was defined as the surface area bearing at least half that of the peak pressure.

This was integrated numerically using Python scipy package (code included in figshare data).
