# Supplementary material for: Lateral pressure equalisation as a principle for designing support surfaces to prevent deep tissue pressure ulcers
Source: PLoS One. 2020 Jan 3;15(1):e0227064. doi: 10.1371/journal.pone.0227064 (PMC6941906; doi:10.1371/journal.pone.0227064)
Supplement: S2 Appendix — (PDF) [file pone.0227064.s002.pdf]

S2 Testing model assumptions

To test the effects of friction on model predictions, we set frictional contact in the 2D model described in the main paper ($\mu = 0.5$). Analysis of von Mises stress predicted in models both with and without friction shows very small differences in peak stresses predicted (Fig 1 a). Peak stress without friction and without lateral pressure applied was 116kPa, while with friction the maximum stress was 115kPa. Lateral pressure had a similar beneficial effect with and without friction (maximum stresses of 31kPa and 40kPa, respectively).

The tissue underlying the ischial tuberosity is patient-specific, and is either muscle or subcutaneous fat. In our 2D models, we assumed that muscle and subcutaneous fat was present. To test the effect of having just subcutanoeus tissue beneath the ischial tuberosity, we adapted our 2D model as shown in Fig 1 b. removing the muscle from under the ischial tuberosity resulted in a change in the location of the peak stress (to the overlying muscle, Fig 1 b). However, the beneficial effects of lateral pressure were still observed, with peak stresses reducing to 40kPa in both models.

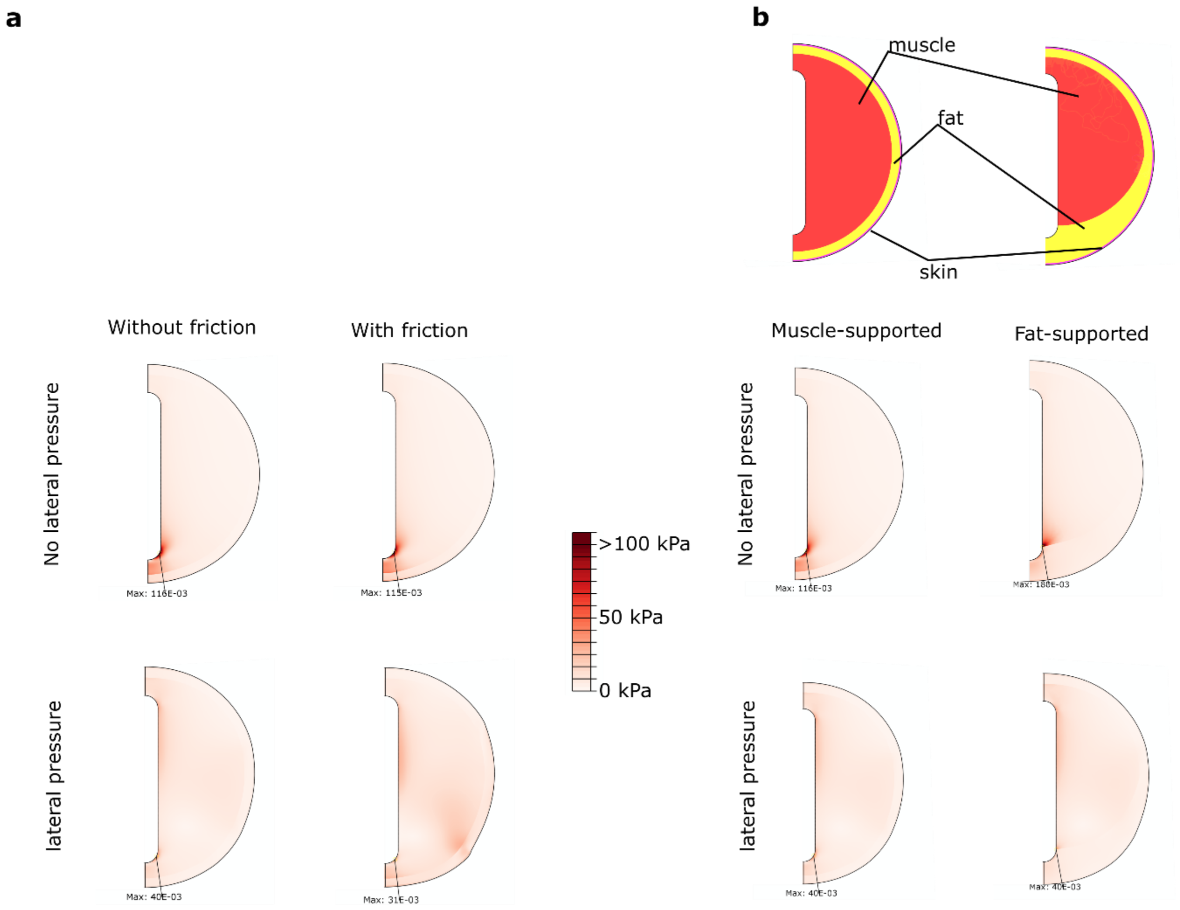

Fig 1. a von Mises stresses in models without (top) and with (bottom) lateral pressure. Original model with frictionless contact (left) versus the same mode with friction (right). **b** schematic showing the original model with underlying muscle tissue and an adapted model in which muscle does not lie under the bone. von Mises stresses shown without lateral pressure (middle) and with lateral pressure (bottom).
