# Supplementary material for: Lateral pressure equalisation as a principle for designing support surfaces to prevent deep tissue pressure ulcers
Source: PLoS One. 2020 Jan 3;15(1):e0227064. doi: 10.1371/journal.pone.0227064 (PMC6941906; doi:10.1371/journal.pone.0227064)
Supplement: Attachment — Submitted filename: responsetoReviewsR1.docx [file pone.0227064.s003.docx]

We wish to thank the reviewers for their valuable critique of our manuscript. Pease see below for our responses to each comment.

Reviewer #1: The paper deals with a basic research regarding the opportunity or not to use lateral pressure to be applied to the patient forced to maintain a sitting position for long periods in order to reduce the pressure at the interface with the pillow and consequently the risk of ulcerations.
The idea is good and its applicability must be verified. The preliminary study is well written and the methods used are appropriate.
Small improvements are required:
- always define all the parameters introduced (for example in table 1 and in the equations)

We have reviewed the manuscript to ensure all parameters are now defined at the point of first instance.

- in paragraph 2.1.2 it is well known that the body mass of the torso + head + upper limbs is equal to 2/3 of the whole body mass not ½

We agree that this 50% value does not represent all upper-body weight. Oomens et al [14] discuss loading boundary conditions in pelvis models. Using literature on experimental pressure-reading, they describe that weight borne by the IT during sitting varies from 18% to 77% of body weight. This variation is due to patient posture, including use of arm and thigh support, and inclination of back support. We chose 50% as a value within the experimental range, while also enabling direct comparison with that previous modelling work [14].

We also argue that because we look at relative differences (between support surfaces), rather than draw conclusions based on absolute values, the choice of load magnitude does not affect our conclusions.

Changes to text:

Section 2.1.2 pg 8:

The proportion of body weight borne by the ischial tuberosities while seated varies from 18% to 77% [14]_._ We estimated the amount of load supported by the pelvis at 400N — representing approximately 50% of the body weight of an 80 kg adult (with each tuberosity bearing 200N) because it is within the range of experimental findings and enables direct comparison with Oomens et al [14].

Discussion pg 25:

A further complexity not accounted for in our model is the posture and secondary supports (arm rests, for example) of the patient, which may affect the loading boundary conditions. For these reasons, we have focused on the relative effects of interventions on stresses and strains, thus making the conclusions robust against the chosen material models and boundary conditions. Using more biofidelic approaches will be a key step in applying the current results in the clinic.

- in paragraph 2.1.2 next to the figure 80 the unit of measurement is missing

Thank you, amended with kPa as units

- in brackets, after the softwares used, it is necessary to mention the company that distributes them and their location

Amended as suggested

- in paragraph 2.3 justify the choices made on the imposed loads.

Our objective for developing this 3D model was to ensure that the conclusions drawn from the axisymmetric model translated to a 3D environment. To achieve this objective, we modelled the same loading regime as the axisymmetric model. We accept, however, that a more biofidelic model – based on realistic load cases including body freedom-of-movement and whole-body simulation – will be required as a next step before translation to the clinic.

Changes to text (the alterations to section 2.1.2 are also relevant here):

Section 2.3 pg 11:

A body force of 200N was applied to the bone nodes. This represents a full body weight of 80 kg, with the assumption that 50% of this travels through the pelvis of a seated individual (as was assumed with the axisymmetric model and is based on Oomens et al. [14], see section 2.1.2). Maintaining this level of loading in the 3D model aids comparison between that and the axisymmetric model.

Reviewer #2: This study sought to present a novel approach to prevent ulcers due to deep tissue pressure. In particular the authors stated that adding a supporting lateral pressure (they called “pressure equalization”) to the classical under-body pressure redistribution approach could provide a more effective solution to reduce deep tissue deformation that can lead to ulcers development. The work is based on Finite Element (FE) Model, which is able to predict the stresses induced on the deep tissue; the authors used this model to assess the differences introduced by the lateral pressure equalization. In particular, they found that managing the ratio of peak lateral pressure to peak under-body pressure and the position of the lateral supports could be used to regulate deep tissue stresses. The authors stated that the overall deformation can be decreased at the induced when the body is suspended in a fluid. The approach can be used to design and assess novel solutions related to supporting devices.
General Comments
The main working hypothesis at the basis of this paper is clearly reported as far as the main objective. The introduction to the approach is clearly reported and the modelling phase well documented. Although the methodology is not that innovative, the application to is indeed original. I guess the audience of PLOS ONE will appreciate this work.
The structure of the article seems to be precise (Abstract, Introduction, Methods [with subheadings], Results, Discussion).
Modelling and data analysis seem to be clearly reported and coherent with the work objectives. Several minor concerns have been already reported to the authors.
The use of the English language seems to be correct.
The references to previous works seem to be precise and up-to-date.
Specific Comments
Abstract
In general this section is ok. Please, could you report also the magnitude of the deformation related to deep tissue and the differences between under-body pressure redistribution and lateral pressure equalization?

Thank you for this suggestion, we have updated the abstract to include magnitudes as well as fold-change.

Changes to text:

Abstract:

A finite element model of the seated pelvis predicts that applying a lateral pressure to the soft tissue reduces peak von Mises stress in the deep tissue by a factor of 2.4relative to a standard cushion (from 113 kPa to 47 kPA) — a greater effect than that achieved by using a more conformable cushion, which reduced von Mises stress to 75 kPa. Combining both a conformable cushion and lateral pressure reduced peak von Mises stresses to 25 kPa.

Introduction
Really well written. You well reported the main clinical problem, the state-of-the-art related to the main provided solutions, your hypothesis and the rationale behind your work. Really appreciated Figure 1.
Methods
• Page 8. Model properties. Oomens’ work is a modelling approach dating back to 2003. Is there any more recent work identifying soft tissues properties and modelling?

Thank you for this comment. We accept that the material properties chose may not be the definitive material models for each of the soft tissues. For example, more complex models that incorporate transverse isotropy of muscle/skin tissue or viscoelasticity may yield more accurate predictions of absolute stress/strain magnitudes. However, our study focuses on relative differences between groups (on different cushions, and with/without lateral pressure).

We argue that this focus on relative differences reduces the sensitivity of our conclusions to specific material models, particularly because our chosen models incorporate the two most important aspects of soft tissue mechanics (large-deformations and material non-linearity). We also note that using these models enabled direct comparison to the study most closely related to our work (Oomens et al [14]).

We also understand that incorporating more accurate material models of soft tissues will be a crucial part of creating more biofidelic models, and that this is a next step on the way to validating our pressure-equalisation approach for the clinic.

Changes to text:

Methods, page 7:

There have been many material models of skeletal muscle [24,25], skin [26], and to a lesser extent fat [27]. However, experimentally-based models that quantify all three tissues together are rarer, making it difficult to combine tissues defined from different experimental setups. In this study, we used the material models based on Oomens et al [14] because it enabled direct comparison with that study, and because all three soft tissues were characterized. Each region was assigned an Ogden hyperelastic material model, and parameter values are listed in Table 1.

Discussion, page 25:

For example, we chose tissue mechanical properties in line with Oomens et al. [14], but there are several published models of soft tissue mechanical properties that vary in complexity [24–27]. This makes conclusions based on absolute stress values difficult. A further complexity not accounted for in our model is the posture and secondary supports (arm rests, for example) of the patient, which may affect the loading boundary conditions. For these reasons, we have focused on the relative effects of interventions on stresses and strains, thus making the conclusions robust against the chosen material models and boundary conditions. Using more biofidelic approaches will be a key step in applying the current results in the clinic.

• Page 11 – 2.1.2 Line 6. I guess that the unit of measurements is missing.

Amended to include kPa as unit.

• Please provide more hypothesis on the choice of not introducing any friction between the support surface and the skin. Is this condition the real one?

We used frictionless contact as a simplification of the real-world situation. To assess the impact of this simplification, we ran a sensitivity study where the effect of including friction on peak stresses and strains was quantified (see S2 Appendix). We found that in our load case, friction had negligible effect on peak stresses. However, we accept that in future studies that aim to simulate more biofidelic load cases, modelling friction effects will become increasingly important. In particular, a model that captured patient posture dynamically would require more accurate contact characterisation.

We also note that our primary focus in this study was the deep tissue (the source of the most dangerous pressure ulcers), while friction tends to induce superficial pressure ulcers.

Changes to text:

Methods page 9:

While this is a simplification of the real-world scenario, a sensitivity study revealed friction to have a negligible effect on model predicitons (S2 Appendix).

• Page 12. Please provide more information about the “adjusting” of data accounting for volume variation. It is not that clear.

Thank you for this comment. We developed an analysis approach to ensure that our conclusions were insensitive to the mesh density. Our aim was to summarise the stress/strain in a tissue. One approach is to simply probe one point near the region of interest. However, this is sensitive to the choice of point. Another approach is to summarise all the points within a region of interest (mean, median, 95 percentile, etc). However, this approach is affected by mesh density, so locations with many small elements contribute more to the summary statistic than locations with fewer large elements. To overcome this limitation, we took a weighted sample of all elements in the region of interest. The sample was weighted by the volume of the element, so an element with double the volume is twice as likely to be sampled.

Changes to text:

Page 10:

To summarise the stresses and strains in the deep tissue, we sampled 1600 elements within a 30mm radius of the ischial tuberosity. Since elements vary considerably in size throughout the region, we weighed our sampling by element volume (IVOL output from ABAQUS). For example, an element with a volume 2V is twice as likely to be sampled as an element with volume V. Weighting the results like this ensures that analyses are independent of mesh density, which varies throughout the model.

• Page 16. Table 2. Please provide information about “[0.5ex]”, present in the first line.

Thank you for spotting this, it is an artefact of a conversion from Latex to MS Word and has been deleted.

Results
Very well reported both graphically and in the main text.
Discussion
In general, this section is ok. Since you reported also the most clinical perspective, what about pressure equalization on vessels? Could you speculate on any main issue?

If we understand your question correctly, you would like us to discuss how our mechanical results translate into improvements to blood perfusion, thereby keeping tissue oxygenated and uninjured. This is a great point, and we have added a paragraph to the discussion.

Changes to text:

Discussion, Page 27:

Our results suggest a novel method for creating a safer mechanical environment. We hypothesize that the reduced deformations created by lateral pressure equalization will translate into better deep tissue blood perfusion and lower risk of deformation-induced cell damage. A key next step will be to test this hypothesis by measuring the physiological response of the soft tissues of seated patients, for example through measuring transcutaneous gas tension [34].
